# The Role of Horizontal Gene Transfer in the Evolution of the Oomycetes

**DOI:** 10.1371/journal.ppat.1004805

**Published:** 2015-05-28

**Authors:** Fiona Savory, Guy Leonard, Thomas A. Richards

**Affiliations:** 1 Biosciences, University of Exeter, Geoffrey Pope Building, Exeter, United Kingdom; 2 Integrated Microbial Biodiversity Program, Canadian Institute for Advanced Research, Toronto, Ontario, Canada; Duke University Medical Center, UNITED STATES

Horizontal gene transfer (HGT) or lateral gene transfer (LGT) involves the transmission of genetic material between distinct evolutionary lineages and can be an important source of biological innovation. For instance, the acquisition of foreign genes can allow recipient organisms to adapt to new lifestyles or to exploit a novel ecological niche, such as a host environment. HGT has long been recognised as an important factor contributing to the evolution of prokaryotic lineages especially in connection to the evolution of pathogencity [1,2]. However, it is becoming increasingly apparent that HGT has also played a role in the evolution of pathogenic traits in eukaryotes [3,4]. Here, we consider how HGT has contributed to genome evolution in the oomycetes.

## What Are Oomycetes?

Oomycetes are eukaryotic microbes that generally grow filamentously and feed osmotrophically by secreting enzymes into the external environment, breaking down complex molecules, and importing nutrients into the cell [5]. These features are the reason they look and behave like fungi (or vice versa depending on your perspective). Indeed, until the use of molecular phylogenies, these microbes were thought to be part of the kingdom Fungi and are still called Pseudofungi by some [6]. However, phylogenetic analysis has shown that they are part of the Stramenopile (Heterokonta) phylum, which includes a range of different forms such as parasites, heterotrophic protists, and both single and multicellular algae, e.g., diatoms and kelps [6,7]. This placement in the tree of life implies that the oomycetes are descended from both a phagotrophic (eukaryotic cell that feeds by engulfing prey microbes into cytoplasmic vesicles for digestion) and photosynthetic ancestor [6]. As such, the evolutionary ancestry of the oomycetes encompasses a radical reconfiguration of lifestyle and trophic mechanism, changing from a cellular form that fixes carbon by photosynthesis and/or digests microbes inside the cell, to a cellular form that processes complex substrates in the extracellular environment in preparation for transportation into the cell.

The class Oomycota encompasses a wide diversity of microbial forms, including free-living saprobes such as *Thraustotheca clavata*, that obtain nutrients from decaying matter, to parasites of plants and animals such as *Phythophthora infestans* and *Saprolegnia parasitica*, respectively [7]. The class contains some of the most important agricultural parasites of plants that cause a range of pathologies, including blights, cankers, wilts, rusts, lesions, and rots, and are estimated to cause an annual loss of over five billion dollars to agriculture in the United States alone [8].

## Has HGT Played a Role in the Evolution of the Oomycetes?

**Fig 1:**
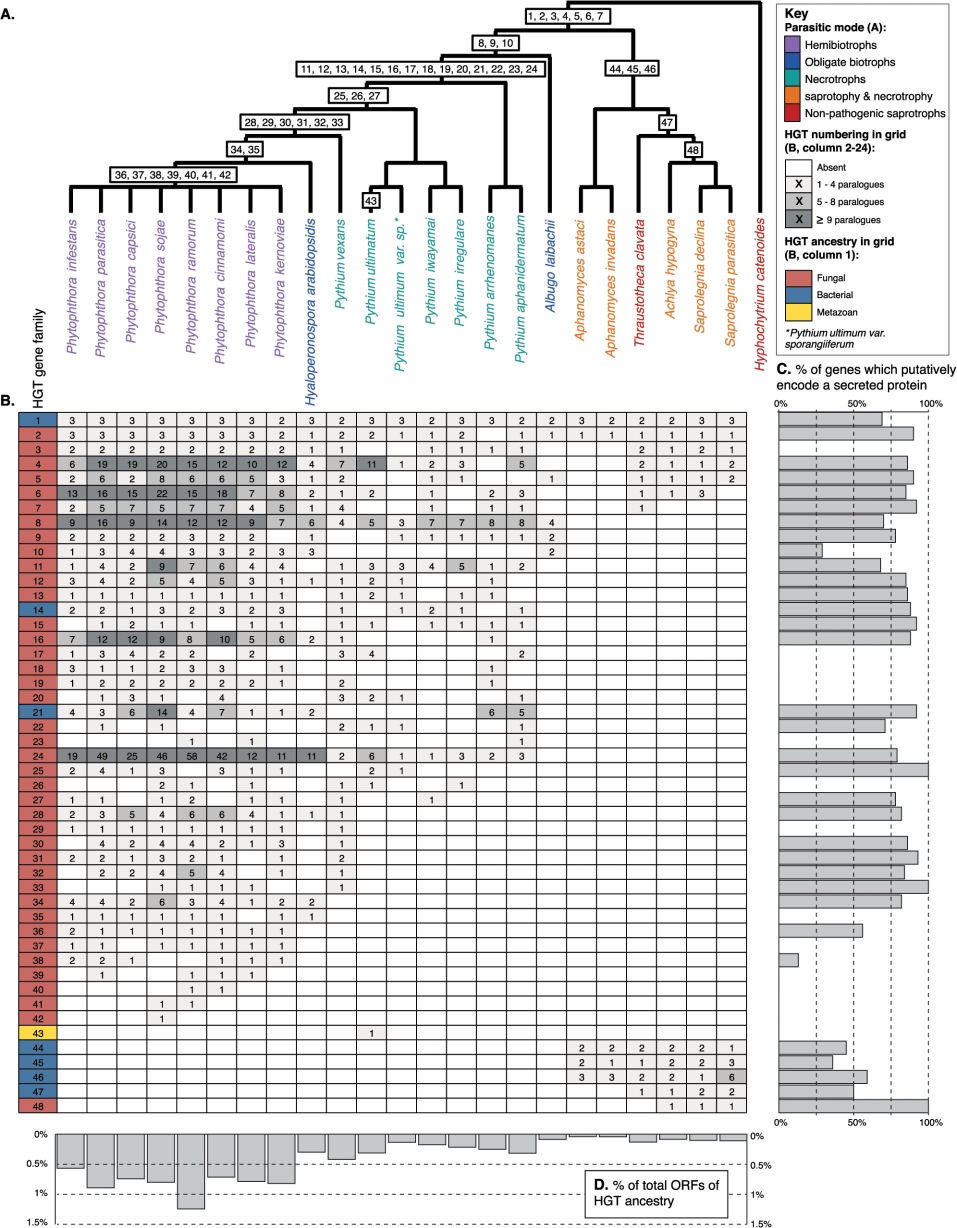
(A) Schematic representation of the phylogeny of the oomycetes with *Hyphochytrium catenoides* as an outgroup. The likely point of acquisition given current genome sampling of each individual HGT is labelled 1–48. See S1 Table for source information and standard of support for each HGT (i.e., phylogeny showing putative HGT gene nested within donor clade or patchy taxonomic distribution of gene family). **(B)** Grid summarising the distribution of 48 HGT gene families and the extent of gene duplication (number of paralogues) for 23 oomycetes. **(C)** The percentage of total gene copies predicted to encode secreted proteins for each HGT family. **(D)** The percentage of total Open Reading Frames (ORFs) from an oomycete genome that have been identified as derived by HGT ancestry.

The oomycetes are one of the better represented classes of microbial eukaryotes in terms of genome sequencing (e.g., [9,10]), with 23 genomes publicly available at the time of composing this summary. This wealth of genome data has led to a number of comparative studies, several of which have identified cases of HGT into the oomycete lineage (e.g., [11–13]). In total, our literature searches identified 48 gene families (each including all paralogues descended from a horizontally acquired ancestral gene) that have been proposed to be transfered into the oomycetes (Fig 1, S1 Table and S2 Table) and that held up to scrutiny when reanalysed using comparative genomic and phylogenetic methods (i.e., oomycete sequences were clearly nested within a donor clade and/or the gene family showed a scattered taxon distribution).

As part of this analysis, we screened the predicted proteomes from 23 currently available oomycete genomes to assess the distribution of these 48 previously identified HGT families. The extent of HGTs identified appears to be highly variable among oomycetes with different lifestyles. For instance, HGT events leading to gene fixation appear to have occurred at the highest frequency in the *Phytophthora* lineage, which includes plant parasitic oomycetes that establish hemibiotrophic interactions with their host plants (Fig 1A, 1B, and 1D, S3 Table), i.e., pathogens that live biotrophically on their hosts then switch to a necrotrophic mode. HGTs were also evident, but to a lesser degree, in the closely related necrotrophic *Pythium* spp. (Fig 1A, 1B and 1D, S3 Table), i.e., pathogens that cause disease by degrading and killing host cells for nutrients. Relatively few HGT genes have been detected in the genomes of obligate biotrophs (i.e., *Hyaloperonospora arabidopsidis* and *Albugo laibachii*—pathogens that feed on living tissue) or *Saprolegniales* spp., which include nonpathogenic saprobes and opportunistic pathogens of plants and animals that obtain nutrients via saprotrophy and/or necrotrophy (Fig 1A).

In only seven cases of HGT could we trace the ancestry of the transfer back to the primary branch of the oomycete phylogeny, prior to the radiation of different lineages (Fig 1A). Likewise, the two secondary branches on the oomycete phylogeny both encompass only three additional HGTs. In contrast, 33 HGTs map within the *Phytophthora* spp., *H*. *arabidopsidis*, and *Pythium* spp. clade. This suggests that HGT had a limited impact on early oomycete evolutionary history and has had a greater impact later within the oomycete radiation, specifically with the radiation of plant parasitic *Pythiaceae* spp. The apparent lack of HGT genes in some oomycetes could potentially reflect an under-representation of available genome sequences for accurate identification of donor lineages [14] and is also biased by the nature of published analyses, which have historically focused on a subset of oomycete genomes and donor groups, at least partly due to the skewed representation of available genome data. Furthermore, consistent with the majority of the oomycete HGTs mapping among the *Phytophthora* hemibiotrophic plant pathogens, many of the putative gene functions are associated with plant pathogenicity (e.g., [4,12,13]).

## Do We See Evidence of Expansion of Horizontally Transferred Genes in the Oomycetes?

The majority of genes that undergo transfer are likely to be selectively neutral or deleterious and therefore lost by drift. However, when acquired genes confer a selective benefit and become fixed in the genomes of a recipient lineage, gene function and dosage are likely to be shaped by selection, leading to improved fitness for the recipient. One mechanism by which this may occur is by gene duplication. For example, if there are constraints on the recipient cell that prevent a horizontally acquired gene from being efficiently expressed, an increase in gene copy number could be selected to allow higher or variant quantities of the corresponding protein to be produced [15]. Moreover, functional divergence of paralogues after duplication can drive the emergence of novel traits (neofunctionalization). We detected evidence of duplication in 38 of the 48 HGTs (Fig 1B). HGT gene family expansion by duplication is particularly evident in the eight hemibiotrophic *Phytophthora* spp. (with a mean of 4.37 gene copies per genome per HGT) compared to the seven *Pythium* spp. that feed by saprotrophy and necrotrophy (mean of 2.12 gene copies per genome per HGT, see also Fig 1D).

## Has HGT Played a Role in the Evolution of the Oomycete Secretome?

The secretome describes all molecules, including proteins, released out of the cell into the external environment. This “molecular characteristic” is of primary importance to how oomycetes make their living, functioning in synthesis of the cell wall, adhesion to host, digestion of host, manipulation of host functions, and nutrient acquisition [5,16]. Genome analysis investigating the nature of secretome diversity has proven important in identifying virulence factors in plant parasitic oomycetes (e.g., [17]). A putative secretome can be identified using bioinformatic methods to identify predicted proteins that carry an N-terminal secretion signal [16,17]. Of the 1,593 predicted proteins that group into the 48 HGT gene families summarised here, 1,152 (73%) are predicted to encode secreted proteins. Remarkably, 33 of the 48 (69%) of the HGT gene families encode putatively secreted proteins (Fig 1C). Taken together, these data demonstrate that HGT has had a major impact upon the evolution of the secretomes of oomycetes, specifically the plant pathogenic *Phytophthora* spp.

## Did HGT Drive Convergent Evolution between Fungi and the Oomycetes?

Given sufficient taxonomic sampling, the direction of gene transfer as well as the approximate origin of a transferred gene within a donor lineage can be inferred from a phylogenetic tree. Reported donors of horizontally acquired genes in the oomycetes include bacteria (e.g., [11,12]), fungi ([11,13]), and animals [18]. However, the majority of fixed HGT genes detected have been acquired from donor genomes arising from within the fungi [11,13]. Indeed, of the 48 oomycete HGTs summarised here, 40 show evidence of fungal origin (Fig 1B).

Oomycetes and fungi are distantly related, but they exhibit similarities in their osmotrophic feeding strategies, life cycles, and filamentous growth characteristics. Many of these characteristics, specifically those associated with the process of infecting plant tissue, were thought to be the product of convergent evolution [19,20]. Yet, annotation of fungal derived oomycete HGT genes has shown that gene transfer has conveyed genes that encode proteins that are predicted to function in processes associated with plant infections such as necrosis and ethylene inducing peptide 1 (NEP1)-like proteins, LysM domain containing proteins, a suite of secreted enzymes that breakdown the structural polysaccharides specific to plant cell walls, and transporters that theoretically allow the parasite to feed on host derived compounds (see [13] for summary of the putative role in pathogenesis). These data demonstrate that HGT was a part of this pattern of convergent evolution.

Taken together, this body of work shows that HGT has played an important role in the evolution of the oomycetes. Although the picture is somewhat biased by the focus of the published studies, this work suggests HGT has been particularly important in the evolution of hemibiotrophic plant pathogenic traits of *Phytophthora* spp. Treatment of *Phytophthora* spp. infections of plants has been hampered historically with limited transferability of antifungal pesticides [8]. Understanding the evolution of oomycete genome content, including the role of HGT from other plant-associated microbes, will open up new avenues for improved pesticide development. As more oomycete genome sequences become available, a broad and systematic analysis of HGT across the whole class will become possible, allowing a complete understanding of the taxonomic distribution and origin of HGTs in the oomycetes.

## Supporting Information

S1 Table List of HGT gene families included in Fig 1 with corresponding annotations, putative pathogenic functions, donor taxa, putative secretory phenotypes, type of phylogenetic support, and references. Representative protein IDs are also provided for each HGT gene family.
(XLSX)

S2 Table List of additional references detailing cases of HGT into the oomycete lineage. (XLSX)

S3 Table Number of paralogues for each HGT gene family included in Fig 1 that were detected in each oomycete species. The total number of paralogues and the percentage of paralogues that were predicted to encode a secreted protein are displayed for each HGT gene family. The total number of HGT genes, mean rates of duplication, and percent of total ORFs that have been horizontally acquired are displayed for each oomycete species considered in the analysis.
(XLSX)
